# The Effects of Fumaria Parviflora L Extract on Chronic Hand Eczema: A Randomized Double-Blind Placebo Controlled Clinical Trial

**Published:** 2011-11-01

**Authors:** F Jowkar, A Jamshidzadeh, A Mirzadeh Yazdi, M Pasalar

**Affiliations:** 1 Department of Dermatology, School of Medicine, Shiraz University of Medical Sciences, Shiraz, Iran; 2 Department of Pharmacology and Toxicology, Faculty of Pharmacy, Shiraz University of Medical Sciences, Shiraz, Iran; 3 School of Medicine, Shiraz University of Medical Sciences, Shiraz, Iran; 4 Research Center for Traditional Medicine and History of Medicine, School of Medicine, Shiraz University of Medical Sciences, Shiraz, Iran

**Keywords:** Eczema, Fumaria parviflora, Fumaric acid

## Abstract

**Background:**

Hand eczema is a common and distressing condition with multiple causes such as atopy, irritant and allergic contact dermatitis. Fumaria parviflora, is known as Shahtareh in Persian, is a folk medicine for eczema. This study aimed to evaluate the effect of alcoholic extract of Fumaria parviflora on hand eczema.

**Methods:**

In a randomized double-blind, placebo-controlled study, 44 patients with hand eczema were randomly assigned to apply 4% cream of Fumaria parviflora or vehicle cream to hand twice daily for 4 weeks.

**Results:**

The reduction of eczema area and severity index score before and two weeks after therapy was statistically significant between vehicle treated and in treated group. Only one patient showed side effects such as erythema and population.

**Conclusion:**

Fumaria parviflora appears to be tolerated by most patients and the findings showed that its extract may be considered as an effective agent for treatment of chronic hand eczema.

## Introduction

Hand eczema is a common and distressing condition, and poses such difficult problems for dermatologists that it deserves separate condition.[1] Patients with hand eczema have multiple causes for their dermatitis such as atopic and irritant or allergic contact dermatitis. Hand eczema represents a major occupational problem and accounts for more than 80% of all occupational dermatitis. Hair dressers, food service workers and health care workers are particularly affected. Both wet and low humidity conditions are risk factors for hand dermatitis. Hard water also increases the incidence of hand dermatitis. Other forms of hand eczema are acute vesiculobullous (pompholyx), chronic vesiculobullous and hyperkeratotic forms.[2] Minor degrees of hand eczema are very common and virtually everyone suffers from mild dryness and chapping at sometime. In most surveys, hand eczema is approximately twice more common in females.[1] Fumaria parviflora L. is known as Shahtareh in Iran. Aqua distillate of aerial parts of Fumaria parviflora (Aragh-e-Shatareh) is used very frequently in different parts of Iran, and is a Persian folk medicine. The aerial parts of plant have been considered to be diuretic, hepatoprotective, laxative, blood purifier and used in liquid form for treatment of scabies, eczema, acne and other skin disorders, externally.[3][4] The present study was undertaken to find out the efficacy of topical cream of the alcoholic extract of this plant on hand eczema.

## Materials and Methods

This randomized double-blind (patient-physician), placebo-controlled study assessed the efficacy of alcoholic extract of Fumaria parviflora (FP) on hand eczema. Randomization was conducted based on block randomization design.

Flowering aerial parts of FP was collected during April 2008 from rural areas around Shiraz, Fars Province, south of Iran, and it was authenticated by a wellknown professor at Botany Department of the Faculty of Science of Shiraz University. The plant was dried at the room temperature. Fifty grams of dried plant powder were macerated in 80% aqueous ethanol (100 ml) at room temperature for 48 hours. The extract was filtered and concentrated under reduced pressure and low temperature (40°C) on a rotary evaporator to dry. The extract yield was 36 mg/gr of dried plant.

Total polyphenols were determined by the Folin– Ciocalteu procedure.[5] Aliquots (0.1 ml) of testsolution were transferred into the test tubes and volumes brought up to 0.5 ml by water. After addition of 0.25 ml Folin–Ciocalteu reagent and 1.25 ml 20% aqueous Na2CO3 solution, tubes were vortexed and absorbance of blue-colored mixtures was recorded after 40 min at 725 nm against blank, containing 0.1 ml of extraction solvent. The amount of total polyphenols was calculated from the calibration curve of routine standard solutions, covering the concentration range between 0.1 and 1.0 mg/ml, and expressed as % (w/w), with regards to dry plant material weight. Stearic acid, paraffin, glycerin, cetyl alcohol, potassium hydroxide, methyl and propyl paraben were used in preparation of 4% FP cream. All of them were of pharmaceutical grade. The stability of the cream was assessed for 3 months.

This study was conducted in autumn 2009, in Shahid Faghihi Skin Clinic attendants, affiliated to Shiraz University of Medical Sciences. Patients were assigned to apply 4% cream of FP or vehicle cream to hands based on block randomization design. Written informed consent was obtained from each patient before initiating study procedures. Healthy patients with hand eczema that did not use topical medication in 2 weeks ago or systemic medication in one month ago were enrolled. Patients were also excluded if they were pregnant or had lactation, or hypersensitivity to test medication which revealed clinically. Patients applied the cream (drug or placebo) to treatment area twice daily for 4 weeks. Efficacy assessments were performed at baseline and two weeks after termination of therapy, according to eczema area and severity index (EASI)[6] (Table 1). A total of 44 patients completed the study. One patient in drug group developed side effect in form of erythema and population after two weeks of therapy that treatment stopped and the patient was excluded from study. Both dermatologist and patients were blind to study groups. Data were recorded by an assessor. Variables were erythema, population, excoriation and lichenification rating each on a scale of 0 (none) to 1 (mild), 2 (moderate), 3 (severe). Demographic and baseline data were summarized for all patients who were matched completely. This clinical trial was registered in the website of Ministry of Health and Medical Education clinical trial center (IRCT, CT-84-2759; IRCT138810303074N1).

**Table 1:** EASI score

**Body region a**	**EASI**
Head and Neck (H)	(E+I+Ex+L)^b^×area score×0.1
Upper limbs (U.L)	(E+I+Ex+L)×area score×0.2
Trunk (T)	(E+I+Ex+L)×area score×0.3
Lower Limbs (LL)	(E+I+Ex+L)×area score×0.4
EASI	Sum^c^ of the above 4 body region scores

^a^ For children aged 0-7 years, proportionate area were: head and neck 20%, upper limbs 20%, trunk 30%, lower limbs 30%

^b^ E: erythema score, I: indurations, population score, Ex: excoriation score, L: lichenification score

^c^ Where area is defined on a 7-point ordinal scale: 0: no eruption, 1: <10%, 2: 10%- 29%, 3: 30%-49%, 4: 50%-69%, 5: 70%-89%, 6: 90%-100%.

The results were shown as mean±standard deviation (SD). For data analysis, SPSS software (Version 13, Chcago, IL, USA) was used and statistical analysis was performed by paired T-test and independent T-test. All statistical comparisons were based on a significant level of p<0.05.

## Results

**Fig. 1:**
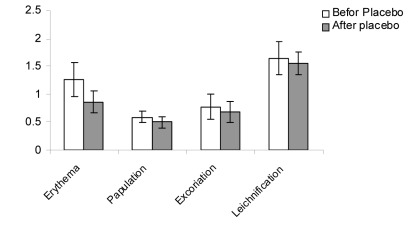
The comparison of clinical manifestation in placebo group before and 2 weeks after treatment. Values are mean±SD.

**Fig. 2:**
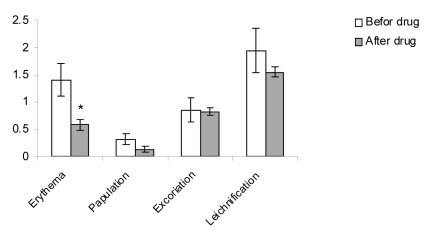
The comparison of clinical manifestation in drug group before and 2 weeks after treatment. Values are mean±SD.

**Fig. 3:**
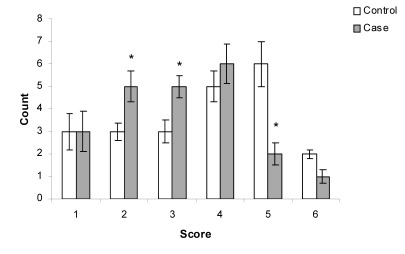
The comparison of disease severity according to EASI score in case and control group two weeks after treatment. Values are mean±SD [*: Significantly different from control group (p<0.05)].

The patient average age was 33.3 years with a range of 13 to 58 years and the majority were female (Table 2). Duration of disease was 46 months in the case and 41 months in the control groups. The reduction of EASI score that was determined by Paired Ttest, was statistically significant in placebo group (pvalue= 0.03). Mean score was 4.3±1.1 before therapy and 3.6±1.6 after therapy (Figure 1). The reduction of EASI score in treated group was statistically significant (p-value≤0.001). Mean score was 4.6±1.5 before and 3.1±1.3 after therapy (Figure 2). By use of independent T-test, statistically significant improvement was observed in EASI score in treated group compared with the placebo group (p value=0.03, mean difference of -0.82, t of -2.22) (Figure 3).

**Table 2:** Patient demographic and baseline characteristic.

N=44
Sex, No. (%)
Male 14 (31.8)
Female 30 (68.2)
Age, Year
Mean 33.3
Range 13-58
Type of eczema, No. (%)
32 ( 72.7 ) irritant contact dermatitis
8 ( 18.4 ) atopic dermatitis
3 ( 6.8 ) allergic contact dermatitis
1 ( 2.2 ) pompholyx

The amount of total polyphenols of our plant was 15 mg (routine equivalents) with regards to 100 gr dry plant material weight.

## Discussion

Super-potent and potent topical steroid agents are first line pharmacological therapies for hand eczema but chronic use of potent fluorinated corticosteroids may be associated with skin atrophy.[2]

Plants are widely used against skin disorders in traditional medicine. Fumaria parviflora plant has been used in the treatment of eczema in the Persian folk medicine. Seven species of the annual plants of genus Fumaria grow in Iran. Fumaria officinalis (FO) is the medicinal species, which is used as a cholagogue. FO (fumitory or earth smoke) is not found in Iran but FP is used instead, in folk medicine. FO is a medicinal plant which has had a role in empirical medicine in numerous countries and has favorable effects on skin complaints.7 We found no previous reports on the effect of Fumaria on eczema, although the antipsoriasis properties of Fumaric acid, one of constituents of Fumitory, have been described.[8] Another study showed the organic and phenolic acids identified in eight Fumaria species have been used for many centuries in folk medicine. The results of this chemotaxonomic investigation showed that the content of Fomaric acid in FP and FO was similar.[9]

Fumaric acid content of FP (from south of Iran) has been determined to be about 0.93% w/w.[10] FP also has fumaric acid esters (FAEs). The effectiveness and safety of FAEs for the treatment of psoriasis has been demonstrated.[11][12] Their mode of action however is poorly understood. FAEs should be considered as drugs showing considerable immunosuppressive efficacy.[10] Mono methylfumarate (MMF) is believed to be the most bioactive metabolite of FAEs.[13] The beneficial effects of FAEs medication are accompanied by a down regulation of type І cytokines production by T-helper lymphocytes.13 Therefore, decreased production of IFN-gamma [14] MMF interfered with monocyte-derived dendritic cells (DCs) differentiation, resulting in impaired maturation of these cells. MMF-incubated iDCs could be maturated by lipopolysaccharide (Lps), these lpsstimulated cells (MMF-DCs) displayed low expression of molecules important for establishing contact with T lymphocytes, therefore MMF-DCs are poor activators of T cells.[13] It has been demonstrated that FAEs have immunomodulatory effects, including inhibition of T lymphocyte proliferation[15][16] as well as inhibition of pro-inflammatory granulocyte cytokines and modulation of monocyte cytokine production. Dimethylfumarate, the main ingredient of FAEs, is a potent inducer of apoptosis in T lymphocyte.[15] FAEs are potential therapy for patients with recalcitrant granulomatous skin disorders and therefore, may be mediated by similar immunomodulatory mechanisms as in psoriasis.16 In the other way, the interaction of trigger factors, keratinocytes and T lymphocytes, seems particularly important in eczema, mostly. In allergic contact dermatitis interaction with the antigen- bearing dendritic cells causes T-lymphocytes differentiation (Th1, Th2) and secreting different pattern of cytokines. Also, in irritant contact dermatitis, the release of inflammatory mediators and cytokines are involved.[1] Therefore, one of the reasons for treatment of hand eczema by FP is existence of FAEs, but other active ingredients must be found in future studies.

Regarding other investigations, the biological activity of Fumaria species is mostly associated with the presence of isoquinoline alkaloids. It might be tempting to speculate that the Fumaria extract has beneficial effect as synthetic fumaric acid esters because possibly the combination of different compound is responsible for the pharmacological effects.[17]

In this study, the results showed that alcoholic FP extract produced improvement in all types of hand eczema in comparison to placebo. In conclusion, the obtained results confirm the presence of immonomodulatory principles in FP, giving a rational support to their use by traditional medicine.
